# Intravenous iron supplementation in heart failure and iron deficiency: an umbrella review of systematic reviews and meta-analyses

**DOI:** 10.3389/fcvm.2026.1902879

**Published:** 2026-10-08

**Authors:** Qiang Li, Yabin Zhou

**Affiliations:** 1 Heilongjiang University of Chinese Medicine, Harbin, Heilongjiang, China; 2 Heilongjiang Academy of Chinese Medicine Sciences, Harbin, Heilongjiang, China

**Keywords:** heart failure, iron deficiency, ferric carboxymaltose, ferric derisomaltose, intravenous iron, umbrella review, meta-analysis, GRADE

## Abstract

**Background:**

Iron deficiency is frequently encountered in patients with heart failure. It is related not only to anemia, but also to exercise intolerance, impaired health status, and repeated hospitalization. Intravenous iron has been evaluated in randomized trials and in many systematic reviews, but the review conclusions are not completely aligned, particularly for mortality and functional outcomes. We therefore reviewed the existing review-level evidence, with particular attention to methodological quality and repeated use of the same primary trials.

**Methods:**

PubMed, Embase, Web of Science, the Cochrane Library, CNKI, and Wanfang were searched from January 2016 to 31 March 2026. Eligible studies were systematic reviews and meta-analyses evaluating iron supplementation in patients with heart failure and iron deficiency. Reviews including mixed iron formulations were considered only when intravenous iron outcomes were separately reported or intravenous iron represented the predominant intervention of interest. AMSTAR2 was used to assess review quality. Trial overlap was examined using the corrected covered area (CCA), and GRADE was used to judge certainty of evidence.

**Results:**

Forty-five eligible reviews were identified, and 18 representative reviews were included in the detailed synthesis. Across the available evidence, intravenous iron showed the most consistent association with reduced heart failure hospitalization, including recurrent hospitalization and composite endpoints combining heart failure hospitalization with cardiovascular death. The randomized evidence was derived predominantly from populations with reduced left ventricular ejection fraction (LVEF), with some trials extending eligibility into the mildly reduced ejection fraction range. Evidence for cardiovascular mortality and all-cause mortality remained inconsistent. Findings for functional capacity and health status were generally favorable but remained uncertain because of heterogeneity and very low certainty evidence, whereas effects on cardiac function were uncertain. No clear increase in serious adverse events was identified with intravenous iron therapy, although the certainty of safety evidence remained limited. According to AMSTAR2, methodological confidence was low in seven reviews and critically low in eleven reviews. The evidence base comprised 23 unique randomized controlled trials with 163 trial occurrences, yielding a CCA of 35.81%, indicating substantial redundancy among representative reviews included in the quantitative synthesis. GRADE certainty was moderate for overall heart failure hospitalization, low for total/recurrent hospitalization, composite hospitalization–cardiovascular death outcomes, all-cause mortality, and serious adverse events, and very low for first hospitalization, cardiovascular mortality, functional outcomes, and cardiac function.

**Conclusions:**

Current review-level evidence, derived predominantly from populations with reduced or mildly reduced LVEF, suggests a potential reduction in heart failure hospitalization. This potential benefit should be interpreted in relation to the patient populations, clinical settings, and iron formulations studied in major randomized trials. The clinical certainty of these findings remains constrained by substantial trial overlap, prominent methodological limitations of the published reviews, and inconsistent outcome definitions. Evidence regarding mortality reduction, functional status, and structural cardiac remodeling remains uncertain and requires cautious interpretation.

## Introduction

Iron deficiency is found in roughly one-third to one-half of patients with heart failure. Even when anemia is not present, it remains clinically important and has been linked to prognosis (1–3). Beyond its classic role in hemoglobin synthesis, iron is a critical cofactor in mitochondrial respiration, oxidative pathways, and cellular energy generation. Depleted cellular iron in heart failure is therefore directly linked to compromised skeletal-muscle function, impaired myocardial bioenergetics, and a subsequent decline in functional capacity (4–7). These changes are reflected clinically in worse health status, heavier symptom burden, repeated hospitalization, and a greater risk of adverse outcomes (2, 8–10). For this reason, recent heart failure guidelines recommend assessment of iron status and intravenous iron replacement in selected patients with confirmed iron deficiency, but these recommendations are phenotype-specific rather than applicable to the entire heart failure spectrum (11–14). The 2022 AHA/ACC/HFSA guideline gives intravenous iron replacement a Class IIa recommendation in patients with heart failure with reduced ejection fraction (HFrEF) and iron deficiency, with or without anemia, to potentially improve functional status and quality of life. The 2023 ESC focused update recommends intravenous iron supplementation in symptomatic patients with HFrEF or heart failure with mildly reduced ejection fraction (HFmrEF) and iron deficiency to alleviate heart failure symptoms and improve quality of life (Class I, Level A), while ferric carboxymaltose or ferric derisomaltose should be considered in these patients to reduce the risk of heart failure hospitalization (Class IIa, Level A). Accordingly, interpretation of intravenous iron evidence requires explicit consideration of left ventricular ejection fraction (LVEF), heart failure phenotype, and clinical setting, rather than simple generalization to all patients with heart failure.

Over the past two decades, evidence for intravenous iron therapy in heart failure has gradually expanded. Early work, including FERRIC-HF and studies of iron sucrose, suggested that patients with heart failure and iron deficiency could have better exercise tolerance, fewer symptoms, and improvement in selected biomarkers after treatment (15–17). Ferric carboxymaltose then became the formulation studied most often. In FAIR-HF, CONFIRM-HF, and EFFECT-HF, improvements were reported in patient-reported outcomes and functional capacity, with an additional signal toward fewer heart failure hospitalizations (18–20).

While recent outcome-driven trials have partially resolved the therapeutic role of intravenous iron, they have also introduced nuance to its clinical interpretation. Specifically, trials such as AFFIRM-AHF, IRONMAN, HEART-FID, and FAIR-HF2 have contributed evidence on the potential for iron supplementation to reduce heart failure-related hospitalizations; however, they have not established a definitive survival benefit (21–24). Such inconsistencies are largely attributable to variations in study populations, specific iron formulations, endpoint criteria, and observation periods across trials. Notably, although ferric carboxymaltose remains the most heavily investigated agent, the accumulation of clinical data on ferric derisomaltose has broadened the therapeutic armamentarium while compounding the complexity of meta-analytic pooling (47). Concomitant with the expanding trial literature, numerous systematic reviews have emerged to synthesize the therapeutic effects of iron therapy in heart failure. Although these reviews span diverse clinical and patient-centered endpoints, their pooled estimates are often confounded by substantial heterogeneity in study selection criteria, statistical modeling, and active treatment definitions (25–29). Several reviews focused exclusively on ferric carboxymaltose, whereas others combined multiple intravenous formulations or compared intravenous and oral iron strategies (30–34).

Despite the proliferation of secondary literature, synthesizing this evidence base remains challenging, as published reviews are frequently confounded by overlapping primary trials and discordant definitions of outcomes, treatment categories, and subgroup cohorts. Consequently, while a superficial consensus across meta-analyses may appear reassuring, it often reflects the redundant compounding of identical randomized trials rather than a truly robust, independent body of evidence. To address this evidentiary redundancy and clarify the true therapeutic signal, we conducted an umbrella review of systematic reviews and meta-analyses investigating intravenous iron therapy in patients with heart failure and concomitant iron deficiency. Beyond summarizing clinical efficacy across key domains—specifically hospitalization, mortality, functional capacity, quality of life, cardiac performance, and safety—we rigorously appraised review-level methodological quality via AMSTAR2, quantified trial redundancy using CCA, and graded the overall certainty of evidence using the GRADE framework.

## Methods

### Design and reporting framework

This umbrella review synthesized evidence from systematic reviews and meta-analyses that evaluated iron supplementation in adults with heart failure and iron deficiency. Study identification, screening, eligibility assessment, and reporting followed the PRISMA 2020 statement (35). The unit of inclusion was the systematic review or meta-analysis, rather than the individual trial. Primary randomized trials were extracted to assess overlap between reviews and to understand repeated contributions of the same evidence, but no patient-level reanalysis was performed.

Eligible populations comprised adults with heart failure and iron deficiency. Heart failure was accepted according to the definitions used in the original systematic reviews, including clinically diagnosed heart failure based on established guideline criteria, documented LVEF phenotypes, including reduced, mildly reduced, or preserved ejection fraction when reported, or other validated clinical classifications. Iron deficiency was accepted according to the criteria reported in the included reviews, most commonly defined as serum ferritin <100 μg/L or ferritin 100–299 μg/L with transferrin saturation <20%. Eligible interventions primarily included intravenous iron formulations, including ferric carboxymaltose, ferric derisomaltose, iron sucrose, and other intravenous iron-carbohydrate complexes. Reviews evaluating mixed iron strategies were considered eligible when intravenous iron represented the primary intervention of interest or when intravenous iron-specific outcomes were reported separately. Oral iron studies were not considered as primary evidence for intravenous iron efficacy but were retained when included within broader iron supplementation reviews to provide contextual information regarding differences between administration routes. Comparators included placebo, usual care, or standard care. Outcomes of interest were heart failure hospitalization, total or recurrent heart failure hospitalization, first heart failure hospitalization, composite endpoints combining heart failure hospitalization with cardiovascular death, cardiovascular mortality, all-cause mortality, 6MWD, KCCQ, NYHA class, LVEF, peak oxygen uptake, serious adverse events, and any adverse events. Studies evaluating oral iron alone without intravenous iron comparison were not considered eligible for the primary effectiveness assessment.

Outcomes were categorized into clinical, functional, patient-reported, cardiac-function, and safety domains. Clinical outcomes included heart failure hospitalization, recurrent or total hospitalization, composite hospitalization–cardiovascular death endpoints, cardiovascular mortality, and all-cause mortality. Functional and patient-reported outcomes included 6MWD, New York Heart Association functional class, peak oxygen uptake, and Kansas City Cardiomyopathy Questionnaire scores. Cardiac-function outcomes included LVEF, whereas safety outcomes included serious adverse events and any adverse events.

We did not restrict this review to mortality outcomes. In heart failure, iron deficiency and iron therapy may be relevant to several areas of care, including hospitalization, symptom burden, exercise capacity, health status, and safety (12, 13, 36). Focusing on a single endpoint would not fully reflect the range of outcomes assessed in recent randomized trials.

Another issue was overlap among studies. Many published reviews were based on a relatively limited set of landmark trials. We therefore included an overlap assessment in the review design, so that agreement across reviews could be interpreted in relation to the amount of shared trial evidence (37, 38).

### Protocol registration

This umbrella review was conducted according to the PRISMA 2020 statement. No protocol was prospectively registered in PROSPERO or another publicly accessible registry. The methodology was developed according to established umbrella review principles, including predefined eligibility criteria, methodological appraisal, overlap assessment, and outcome-level certainty evaluation (48).

### Search strategy and eligibility criteria

We searched PubMed, Embase, Web of Science, the Cochrane Library, CNKI, and Wanfang. The search covered publications from January 2016 to 31 March 2026 to capture contemporary systematic reviews and meta-analyses developed after the expansion of large randomized trials of intravenous iron therapy in heart failure. The final search was conducted on 31 March 2026.

Search strategies combined controlled vocabulary and free-text terms related to heart failure, iron deficiency, intravenous iron, ferric carboxymaltose, ferric derisomaltose, iron sucrose, iron supplementation, systematic review, and meta-analysis. Search syntax was adapted according to the indexing requirements of each database. The complete search strategies for all databases are provided in Supplementary Table 4.

No language restrictions were applied. Duplicate records were removed using reference-management software before title and abstract screening. Detailed search strategies, database-specific terms, and screening procedures are presented in the Supplementary Material.

Eligible reviews were systematic reviews with quantitative synthesis, including conventional meta-analyses, network meta-analyses, and subgroup meta-analyses. They had to evaluate iron supplementation in adults with heart failure and iron deficiency. To be included, a review also had to report at least one outcome relevant to this umbrella review, such as clinical outcomes, functional outcomes, patient-reported outcomes, cardiac-function measures, biomarkers, or safety outcomes.

Reviews were excluded if they focused on populations without heart failure, evaluated interventions unrelated to iron supplementation, examined anemia in the absence of both heart failure and iron deficiency, or discussed only mechanistic or biomarker associations without treatment outcomes. Non-systematic evidence sources were also excluded, including protocols, narrative reviews, scoping reviews, guidelines, editorials, letters, commentaries, conference materials, case reports, animal studies, and expert opinions. In addition, we excluded individual patient data meta-analyses, pooled analyses that were not conducted as systematic reviews, and trial-level pooled analyses, because the unit of inclusion was the systematic review or meta-analysis.

When more than one version of a review was available, we retained the most complete report. This decision was based on the completeness of methodological reporting, availability of outcome data, description of the included trials, and clarity of population and intervention details. Conference abstracts were not eligible because they did not provide enough information for methodological quality assessment, overlap mapping, or certainty-of-evidence assessment.

### Study selection

Records were de-duplicated before screening. Titles and abstracts were screened against predefined eligibility criteria, and potentially relevant reports underwent full-text assessment. When full-text PDFs were unavailable, eligibility was evaluated using alternative sources, including abstracts, PubMed Central records, bibliographic databases, publisher webpages, and other accessible sources. Reports were not excluded solely because a downloadable PDF could not be obtained. Two authors independently participated in the study selection and data extraction process. QL conducted the primary screening, extraction, methodological assessment, and evidence synthesis, while YZ independently cross-checked eligibility decisions, extracted information, reviewed methodological assessments, and critically evaluated the certainty assessment and interpretation of findings. Any disagreements were resolved through discussion and consensus.

### Prioritization of representative reviews

Although 45 systematic reviews and meta-analyses fulfilled the predefined eligibility criteria, many reviews included the same randomized controlled trials and addressed highly overlapping clinical questions. Directly synthesizing all eligible reviews in equal detail would therefore risk duplicating evidence and artificially inflating the apparent evidence base. To reduce redundant evidence contribution, 18 representative reviews were prioritized for detailed methodological appraisal, overlap assessment, GRADE evaluation, and evidence synthesis. The remaining 27 eligible reviews were retained to describe the broader review-level evidence context and to support concordance assessment.

Prioritization was based on predefined hierarchical criteria. First, reviews most closely matching the predefined population, intervention, comparator, and outcome framework were prioritized. Second, priority was given to reviews providing clear, extractable, outcome-specific quantitative estimates. Third, when overlap existed, reviews incorporating contemporary randomized controlled trial evidence, including newer landmark trials, were prioritized. Fourth, among otherwise comparable reviews, preference was given to reviews providing broader and less duplicated evidence coverage.

When multiple reviews fulfilled similar criteria, the review providing the most comprehensive and interpretable evidence synthesis was selected. Methodological reporting completeness was considered only to ensure transparency and reproducibility of assessment, rather than as a measure of review quality or treatment effect. AMSTAR2 methodological confidence was not used as a selection criterion, and reviews were not prioritized according to favorable or unfavorable findings. The prioritization process and rationale for individual non-prioritized reviews are provided in Supplementary Table 5. Reviews were classified according to their contribution to the umbrella review as quantitative synthesis, outcome-specific synthesis, or contextual evidence.

### Data extraction

For each included review, data were extracted on publication characteristics, review methodology, study populations, interventions, comparators, outcomes, and principal findings. Extracted variables included first author, publication year, journal, review type, number of included studies and randomized controlled trials, total participants, intervention and comparator characteristics, follow-up duration when available, outcome measures, effect estimates, heterogeneity statistics, risk-of-bias assessment methods, publication-bias assessment, funding, and conflicts of interest. Heart failure phenotype, LVEF eligibility criteria, and the acute vs. chronic or stable clinical setting were additionally summarized from the included reviews and underlying landmark randomized trials where clearly reported, to inform interpretation of evidence generalizability. Data extraction was based primarily on the main text, tables, Supplementary Materials, and forest plots. Abstract information was used only when full-text information was unavailable or incomplete. Extracted data were cross-checked against the source reviews by the second author, and discrepancies were resolved through discussion and consensus.

To improve consistency across reviews, participant counts were recorded as total participants, while subgroup-specific sample sizes were documented separately when relevant. Review types were categorized as meta-analysis, systematic review and meta-analysis, network meta-analysis, or subgroup meta-analysis. Interventions were classified as intravenous iron, ferric carboxymaltose, ferric derisomaltose, oral iron, or mixed iron formulations.

For outcomes reported in multiple reviews, preference was given to estimates that most closely matched the predefined outcome definitions. Recurrent-event outcomes were extracted separately from first-event outcomes whenever this distinction was reported. When multiple estimates for the same outcome were available within a review, the primary result identified by the review authors was selected, and supplementary analyses were recorded separately. This approach aimed to minimize misclassification of distinct outcomes such as first hospitalization, recurrent hospitalization, total hospitalization, and composite hospitalization–mortality endpoints. Representative estimates were selected according to predefined criteria, including alignment with prespecified outcome definitions, clinical interpretability, inclusion of contemporary randomized evidence, and avoidance of duplicate representation of overlapping evidence.

### Methodological quality assessment

The methodological quality of included reviews was evaluated using AMSTAR2 (39). Each review was assessed across the 16 AMSTAR2 items, with emphasis on the critical domains identified by the instrument. Overall confidence was classified according to AMSTAR2 recommendations as high, moderate, low, or critically low. AMSTAR2 ratings were used to characterize the quality of the review evidence and were not converted into numerical scores.

### Overlap assessment

To assess overlap among reviews, all primary randomized trials included in the representative reviews were identified and mapped into a citation matrix. Trial names were harmonized when different reviews used alternative naming conventions for the same study. The resulting matrix was used to determine the extent to which reviews relied on shared primary evidence.

Overlap was quantified using CCA, calculated as (Nr−Ns)/[(Nc × Ns)−Ns], where Nc denotes the number of reviews, Ns the number of unique primary studies, and Nr the total number of study occurrences within the matrix (37, 38). CCA values were interpreted using established thresholds for slight, moderate, high, and very high overlap.

CCA was calculated among the 18 representative reviews included in the quantitative synthesis. Therefore, this estimate reflects redundancy within the prioritized evidence set rather than overlap across all 45 eligible reviews. The overlap analysis was not used as a criterion to select representative reviews; instead, it was performed after prioritization to characterize the degree of redundancy among the reviews contributing to the primary synthesis.

### Certainty of evidence

GRADE was used to assess certainty for each outcome (40). Evidence derived from randomized controlled trials was initially considered high certainty and was downgraded according to risk of bias, inconsistency, indirectness, imprecision, and publication bias.

In this umbrella review, AMSTAR2 methodological confidence and GRADE certainty were considered complementary but distinct assessments. AMSTAR2 evaluates the methodological rigor of systematic reviews, whereas GRADE evaluates the certainty of evidence supporting individual clinical outcomes. Therefore, low AMSTAR2 confidence did not automatically result in downgrading of all outcomes to low or very low certainty. Instead, AMSTAR2 limitations informed interpretation of the overall evidence context.

For outcomes with consistent effects across reviews, support from larger randomized trials, acceptable precision, and clinically coherent findings, certainty was maintained when the GRADE domains did not justify further downgrading. Conversely, certainty was downgraded when estimates were affected by substantial heterogeneity, indirect comparisons, imprecision, limited trial numbers, or concerns regarding publication bias.

Final certainty categories were classified as high, moderate, low, or very low. Certainty judgments were performed for heart failure hospitalization, total heart failure hospitalization, first heart failure hospitalization, composite heart failure hospitalization plus cardiovascular death, cardiovascular mortality, all-cause mortality, 6MWD, KCCQ, LVEF, and serious adverse events.

### Evidence map

We also constructed an evidence map to present the findings more clearly across outcomes. The map summarized two aspects at the same time: the direction of effect and the certainty of evidence. Outcomes were grouped as beneficial, no clear effect, uncertain, or potentially harmful. Certainty was classified using GRADE. We also included information on the amount of available evidence, because a favorable direction of effect is easier to interpret when it is supported by a less sparse evidence base.

When participant numbers were not reported for a specific outcome, we used the closest available review-level participant count as a proxy and recorded this decision.

## Results

### Search results and selection of reviews

The database search identified 515 records: 72 from PubMed, 190 from Embase, 117 from Web of Science, 5 from the Cochrane Library, 83 from CNKI, and 48 from Wanfang. After removal of 109 duplicate records, 406 records underwent title and abstract screening. Of these, 316 were excluded, and 90 reports were assessed for eligibility. Forty-five systematic reviews and meta-analyses fulfilled the predefined eligibility criteria.

Because multiple eligible reviews addressed similar clinical questions with substantial overlap in randomized trial populations, a predefined prioritization strategy was applied to identify representative reviews for detailed methodological appraisal, overlap assessment, GRADE evaluation, and evidence synthesis. Eighteen representative reviews were selected based on methodological reporting transparency, availability of extractable outcome data, relevance to the PICO framework, recency of evidence, and minimization of redundant synthesis. The remaining eligible reviews were retained as part of the overall evidence base but were not included in the detailed synthesis because they provided limited additional independent information beyond the prioritized reviews. The selection process is summarized in the PRISMA 2020 flow diagram (Figure 1).

**Figure 1:**
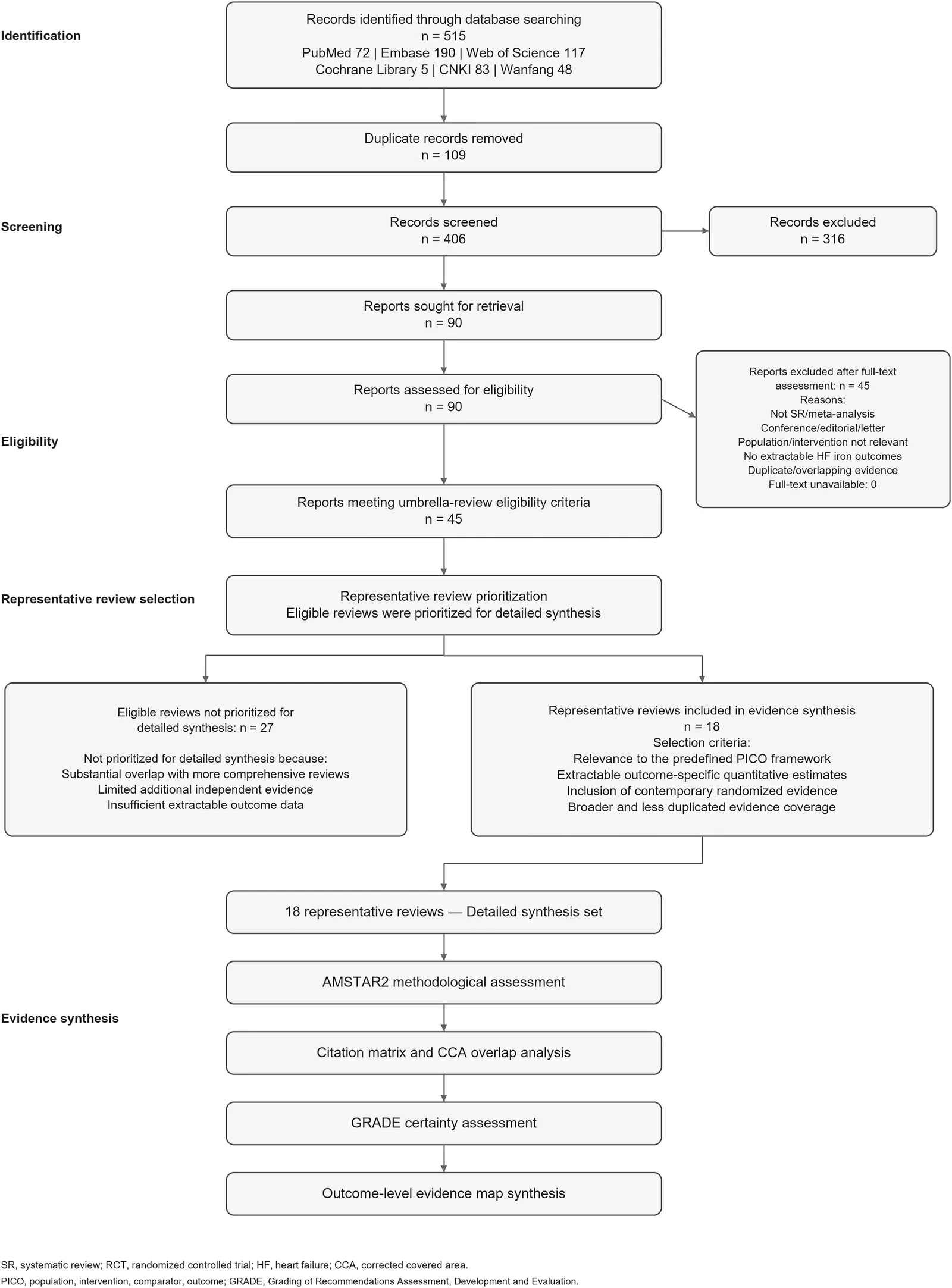
PRISMA 2020 flow diagram for the umbrella review. The study selection process was conducted according to the PRISMA 2020 statement.

### Characteristics of included reviews

The 18 representative reviews were published between 2016 and 2026. Most were systematic reviews with meta-analysis; one network meta-analysis and one subgroup meta-analysis were included. The number of included studies ranged from 4 to 14, and most reviews primarily synthesized randomized controlled trials. Table 1 summarizes the characteristics of included reviews, including population, intervention, comparator, outcomes, and AMSTAR2 confidence rating.

**Table 1:** Characteristics of representative systematic reviews included in the umbrella review.

Author/year	Review design	Population	Intervention	Comparator	Included trials	Sample size	Main outcomes	Search date	AMSTAR2 confidence	Iron intervention classification	Contribution to umbrella review
Abu-Dayyeh 2023	Network meta-analysis	Adults with heart failure and iron deficiency	Mixed iron formulations	Placebo, usual care, or standard care	5 RCTs	NR	6MWD; KCCQ	NR	Critically low	Mixed intravenous formulations	Outcome-specific synthesis
Vukadinovic 2023	Systematic review and meta-analysis	Adults with heart failure and iron deficiency; 3,258 in sensitivity analysis	Mixed iron formulations	Placebo, usual care, or standard care	2 main trials; 7 in sensitivity analysis RCTs	2,250	HFH; CVM; ACM	NR	Low	Mixed iron strategies (IV-specific outcomes extracted)	Contextual evidence
Graham 2023 (27)	Systematic review and meta-analysis	Adults with heart failure and iron deficiency	Intravenous iron	Placebo, usual care, or standard care	10 RCTs	3,373	HFH; CVM; ACM	NR	Critically low	Intravenous iron only	Included in quantitative synthesis
Taha 2024 (32)	Systematic review and meta-analysis	Adults with heart failure and iron deficiency	Ferric carboxymaltose (FCM)	Placebo, usual care, or standard care	10 RCTs	6,271	HFH; CVM; ACM; 6MWD; KCCQ	NR	Critically low	Ferric carboxymaltose only	Included in quantitative synthesis
Rangwala 2024 (33)	Systematic review and meta-analysis	Adults with heart failure and iron deficiency	Ferric carboxymaltose (FCM)	Placebo, usual care, or standard care	8 RCTs	5,229	HFH; CVM; ACM; Safety	NR	Critically low	Ferric carboxymaltose only	Included in quantitative synthesis
Wang 2024 (56)	Systematic review and meta-analysis	Adults with heart failure and iron deficiency	Intravenous iron	Placebo, usual care, or standard care	11 RCTs	6,511	HFH; CVM; ACM; Safety	NR	Critically low	Intravenous iron only	Included in quantitative synthesis
Marques 2024 (49)	Subgroup meta-analysis	Adults with heart failure and iron deficiency; TSAT subgroup: 2,592; ferritin subgroup: 4,114	Intravenous iron	Placebo, usual care, or standard care	4 RCTs	NR	HFH; CVM	NR	Low	Intravenous iron only	Outcome-specific synthesis
Mhanna 2024 (42)	Systematic review and meta-analysis	Adults with heart failure and iron deficiency	Intravenous iron	Placebo, usual care, or standard care	14 RCTs	6,614	HFH; CVM; ACM; 6MWD; KCCQ; LVEF; Safety	NR	Critically low	Intravenous iron only	Included in quantitative synthesis
Pintaningrum 2024 (53)	Systematic review and meta-analysis	Adults with heart failure and iron deficiency; Participants from 21 countries	Mixed iron formulations	Placebo, usual care, or standard care	7 RCTs	1,009	HFH; ACM; 6MWD	NR	Critically low	Mixed iron strategies (IV-specific outcomes extracted)	Contextual evidence
Martens 2024 (10,28)	Systematic review and meta-analysis	Adults with heart failure and iron deficiency	Intravenous iron	Placebo, usual care, or standard care	14 RCTs	6,624	HFH; CVM	NR	Critically low	Intravenous iron only	Included in quantitative synthesis
Padda 2024 (34)	Systematic review and meta-analysis	Adults with heart failure and iron deficiency	Ferric carboxymaltose (FCM)	Placebo, usual care, or standard care	6 RCTs	5,132	HFH; CVM; ACM; 6MWD; KCCQ; Safety	NR	Low	Ferric carboxymaltose only	Included in quantitative synthesis
Shalabi 2025 (54)	Systematic review and meta-analysis	Adults with heart failure and iron deficiency	Ferric carboxymaltose (FCM)	Placebo, usual care, or standard care	9 RCTs	7,491	HFH; CVM; ACM; 6MWD; KCCQ; Safety	NR	Low	Ferric carboxymaltose only	Included in quantitative synthesis
Ahmed 2025 (46)	Systematic review and meta-analysis	Adults with heart failure and iron deficiency	Intravenous iron	Placebo, usual care, or standard care	14 RCTs	6,651	HFH; CVM; ACM; 6MWD; LVEF; Safety	NR	Low	Intravenous iron only	Included in quantitative synthesis
Mohamed 2026 (43)	Systematic review and meta-analysis	Adults with heart failure and iron deficiency	Ferric carboxymaltose (FCM)	Placebo, usual care, or standard care	9 RCTs	6,405	HFH; CVM; ACM; 6MWD; KCCQ	NR	Low	Ferric carboxymaltose only	Included in quantitative synthesis
Ahmed 2026 (29)	Systematic review and meta-analysis	Adults with heart failure and iron deficiency	Ferric carboxymaltose (FCM)	Placebo, usual care, or standard care	11 RCTs	6,493	HFH; CVM; ACM; 6MWD; KCCQ; Safety	NR	Critically low	Ferric carboxymaltose only	Included in quantitative synthesis
Qian 2016 (25)	Systematic review and meta-analysis	Adults with heart failure and iron deficiency	Mixed iron formulations	Placebo, usual care, or standard care	5 RCTs	907	HFH; ACM; Safety	NR	Critically low	Mixed iron strategies (IV-specific outcomes extracted)	Contextual evidence
Mototani 2023 (51)	Meta-analysis	Adults with heart failure and iron deficiency	Intravenous iron	Placebo, usual care, or standard care	8 RCTs	3,329	HFH; CVM; ACM	NR	Critically low	Intravenous iron only	Outcome-specific synthesis
Tanashat 2024 (41)	Systematic review and meta-analysis	Adults with heart failure and iron deficiency	Intravenous iron	Placebo, usual care, or standard care	14 RCTs	6,626	HFH; CVM; ACM; 6MWD; Safety	NR	Low	Intravenous iron only	Outcome-specific synthesis

Total heart failure hospitalization, composite heart failure hospitalization plus cardiovascular death, all-cause mortality, and serious adverse events were rated as low certainty. First heart failure hospitalization, cardiovascular mortality, 6MWD, KCCQ, and LVEF were rated as very low certainty because of greater inconsistency, imprecision, indirectness, or limited available evidence. No outcome achieved high certainty.

Most representative reviews evaluated intravenous iron therapy, particularly ferric carboxymaltose. Some reviews incorporated mixed iron formulations or included oral iron comparisons; these were interpreted primarily as contextual evidence because the primary clinical question of this umbrella review concerned intravenous iron therapy. Reviews evaluating mixed strategies were retained when intravenous iron represented the predominant intervention or when intravenous iron-specific outcomes were reported separately. Later reviews incorporated contemporary trials including AFFIRM-AHF, IRONMAN, HEART-FID, and FAIR-HF2, resulting in greater emphasis on hospitalization outcomes, composite clinical endpoints, and mortality.

### AMSTAR2 methodological confidence

The methodological confidence of included reviews was generally limited. Seven reviews were rated as low confidence and eleven as critically low confidence according to AMSTAR2. No review achieved moderate or high confidence.

The most frequent methodological limitations included absence of prospectively registered protocols, incomplete reporting of excluded full-text studies with reasons, inadequate duplicate processes for study selection or data extraction, limited incorporation of risk-of-bias assessments into interpretation, and incomplete assessment of publication bias.

### Overlap among primary randomized trials

The 18 representative reviews included 23 unique randomized controlled trials. These primary trials appeared 163 times across the citation matrix, resulting in a CCA of 35.81%, indicating substantial overlap among representative reviews included in the quantitative synthesis.

Frequently reused trials mainly included intravenous iron studies, such as FAIR-HF, CONFIRM-HF, EFFECT-HF, AFFIRM-AHF, IRONMAN, HEART-FID, FAIR-HF2, FERRIC-HF, Myocardial-IRON (52), and IRON-CRT (50), and earlier iron sucrose studies. IRONOUT-HF was identified in some mixed iron supplementation reviews and was retained only for overlap characterization; it was not considered as evidence for intravenous iron efficacy interpretation. Trial names and aliases were harmonized before calculation. The final overlap parameters were Nc = 18, Ns = 23, and Nr = 163. The citation overlap matrix is shown in Figure 2.

**Figure 2:**
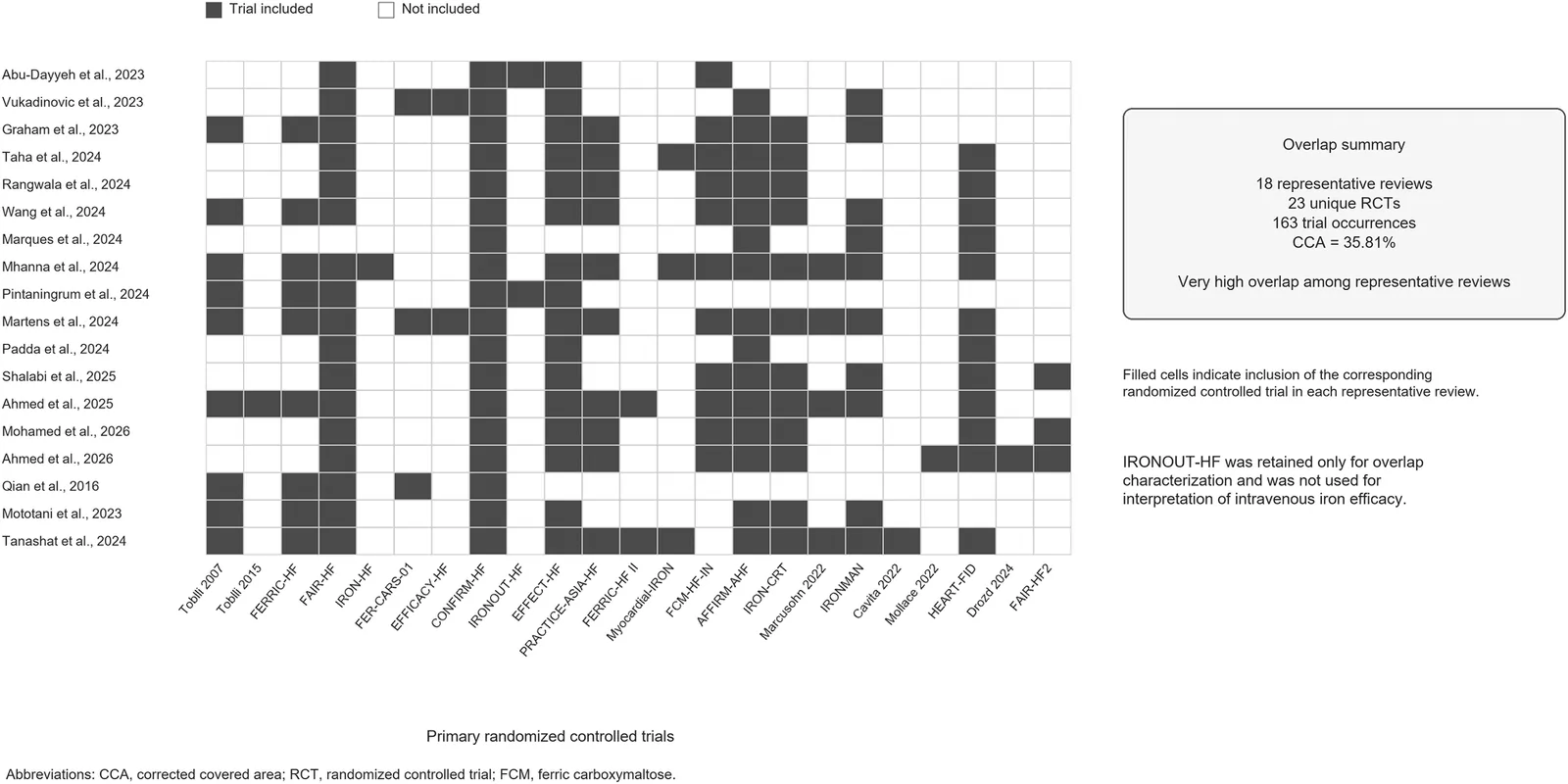
Citation overlap matrix of randomized controlled trials across representative reviews. CCA, corrected covered area; RCT, randomized controlled trial.

### Heart failure phenotypes represented in the randomized evidence base

The randomized evidence underlying the included reviews was derived predominantly from patients with reduced LVEF, with several contemporary trials extending eligibility into the mildly reduced ejection fraction range. Stable chronic heart failure trials such as FAIR-HF, CONFIRM-HF, EFFECT-HF, FERRIC-HF, IRONMAN, HEART-FID, FAIR-HF2, Myocardial-IRON, and IRON-CRT predominantly enrolled patients with reduced LVEF, although several used eligibility thresholds that extended into the contemporary mildly reduced EF range. AFFIRM-AHF evaluated patients stabilized after an acute heart failure hospitalization and used a broader LVEF eligibility threshold below 50%, thereby representing a post-acute population extending beyond strictly reduced ejection fraction. Smaller earlier iron sucrose studies also contributed to the evidence base but were less consistently reported across reviews. Evidence specifically derived from heart failure with preserved ejection fraction (HFpEF) populations was limited. Accordingly, the review-level evidence should not be interpreted as representing the full spectrum of heart failure phenotypes.

Because the remaining 27 eligible reviews were not included in the quantitative synthesis due to limited additional independent information, CCA was interpreted as a measure of overlap within the synthesized evidence base rather than the entire eligible review landscape.

### Sensitivity analysis of evidence direction across all eligible reviews

A qualitative concordance assessment was performed by comparing conclusions from the 18 prioritized reviews with those reported in the remaining 27 eligible reviews. The overall direction of evidence was consistent: intravenous iron was generally associated with reduced heart failure hospitalization, while effects on mortality and functional outcomes remained uncertain. No additional eligible review identified findings that would materially change the direction or interpretation of the main conclusions.

Filled cells indicate inclusion of the corresponding randomized controlled trial in each systematic review. The corrected covered area (CCA) was used to quantify overlap among reviews. The matrix includes only the 18 representative reviews selected for detailed synthesis.

### Heart failure hospitalization

Review-level findings for heart failure hospitalization were generally consistent with a favorable association of intravenous iron therapy, though the magnitude of the clinical benefit varied depending on the specific endpoint definition and statistical approach utilized by the authors. To maintain clinical and methodological rigor, we analyzed these findings according to three distinct estimands: first-event hospitalization (reflecting time-to-first clinical worsening), total or recurrent hospitalization (reflecting overall disease burden), and composite clinical endpoints combining hospitalization with cardiovascular mortality. For first-event heart failure hospitalization, the clinical signal was generally favorable but showed some statistical variability across the reviews. Graham et al. reported a reduction in first heart failure hospitalization with a risk ratio of 0.83 (95% CI, 0.71–0.97). Martens et al. reported a favorable effect for total heart failure hospitalization, with a rate ratio of 0.81 (95% CI, 0.74–0.89). Other reviews, including Vukadinović et al., Rangwala et al., Shalabi et al., and Ahmed et al., also reported directionally favorable effects for hospitalization-related outcomes.

Total or recurrent heart failure hospitalization outcomes showed favorable estimates, although interpretation was limited by heterogeneity and low certainty evidence. Martens et al. reported a rate ratio of 0.739 (95% CI, 0.661–0.827), while Padda et al. reported a pooled estimate of 0.59 (95% CI, 0.40–0.88). However, heterogeneity was substantial in some analyses, with I² values approaching 75%–78%. Evidence concerning first heart failure hospitalization was less consistent. While Ahmed et al. (29) demonstrated a significant reduction with an odds ratio of 0.855 (95% CI, 0.744–0.983), alternative analyses—such as the review by Tanashat et al. (41)—yielded less certain estimates with confidence intervals crossing the null. Conversely, composite endpoints combining heart failure hospitalization with cardiovascular death consistently favored intravenous iron. Reviews by Taha et al. (32), Mhanna et al. (42), and Mohamed et al. (43) reported pooled effect estimates ranging from 0.79 to 0.89, though differences in outcome definitions and follow-up durations precluded direct statistical comparison. Within the consolidated evidence map, overall heart failure hospitalization was categorized as beneficial, whereas first-event hospitalization remained uncertain because of limited and inconsistent evidence. According to the GRADE assessment, the certainty of evidence was moderate for overall heart failure hospitalization, low for both recurrent/total hospitalization and composite endpoints, and very low for first heart failure hospitalization.

### Mortality outcomes

#### Cardiovascular mortality

Evidence for cardiovascular mortality remained inconclusive. Graham et al., Martens et al., Shalabi et al., and Ahmed et al. reported estimates generally in the range of risk ratios or odds ratios of approximately 0.87–0.90. However, several analyses had confidence intervals crossing the null, and statistical significance was inconsistent across reviews.

Cardiovascular mortality was therefore classified as uncertain in the evidence map. GRADE certainty was rated as very low because of inconsistency, imprecision, and methodological limitations of the available review-level evidence.

#### All-cause mortality

Evidence for all-cause mortality did not demonstrate a reliable treatment effect. Qian et al., Vukadinović et al., Wang et al., and Mhanna et al. reported estimates generally close to the null, with risk ratios or odds ratios approximately between 0.90 and 0.95. Earlier reviews were limited by smaller sample sizes and wider confidence intervals.

The direction of effect was classified as no clear effect in the evidence map. GRADE certainty was rated as low because of imprecision and limitations of the contributing reviews.

### Functional capacity, health status, and cardiac function

#### Six-minute walk test

Effects on exercise capacity were heterogeneous. Taha et al. and Pintaningrum and Yunandari reported improvements in 6MWD of approximately 29–31 m, whereas Padda et al. reported a smaller mean difference of 14.03 m with confidence intervals crossing the null.

The direction of effect for 6MWD remained uncertain despite favorable estimates reported in some reviews, because of substantial heterogeneity and very low certainty evidence.

#### KCCQ

KCCQ outcomes were inconsistent across reviews. Taha et al. and Padda et al. reported variable effects, with some analyses suggesting improvement and others showing no statistically significant difference. High heterogeneity and differences in patient populations, follow-up duration, and treatment protocols limited interpretation.

KCCQ effects remained uncertain, with some analyses suggesting improvement whereas others showed no statistically significant difference. Interpretation was limited by inconsistent estimates, heterogeneity, and very low certainty evidence.

#### LVEF

Evidence for LVEF improvement was also uncertain. Mhanna et al. and Padda et al. reported mean differences of approximately 2.7% and 4.54%, respectively; however, confidence intervals were imprecise and clinical significance remained uncertain.

LVEF was classified as uncertain with very low GRADE certainty.

#### Safety outcomes

No clear increase in serious adverse events was identified across available reviews. Rangwala et al. reported a risk ratio of 0.98 (95% CI, 0.93–1.03), while Padda et al. reported an estimate of 0.73 (95% CI, 0.49–1.10).

However, definitions and reporting methods for serious adverse events varied across reviews, and heterogeneity was moderate to high in some analyses. Serious adverse events were classified as showing no clear effect, with low GRADE certainty.

Any adverse events were reported less consistently. Several reviews summarized adverse events narratively or used trial-specific definitions, limiting direct comparison. Infusion reactions, infection-related outcomes, hypotension, and gastrointestinal events were not uniformly reported across iron formulations. Ferric carboxymaltose-associated hypophosphatemia and hypersensitivity reactions were also not consistently captured across reviews; therefore, uncommon or formulation-specific harms could not be fully excluded.

#### GRADE summary and evidence map

GRADE certainty varied across outcomes. Heart failure hospitalization was rated as moderate certainty because the direction of effect was consistent across multiple reviews, supported by contemporary randomized trial evidence, and generally associated with clinically meaningful estimates with acceptable precision. However, certainty was not rated as high because methodological limitations of the contributing reviews, substantial overlap among primary trials, and variability in hospitalization definitions remained important sources of uncertainty. This moderate certainty reflects consistency and coherence at the outcome level rather than high methodological confidence of the contributing systematic reviews.

The evidence map summarizes the direction of effect and certainty of evidence across major outcomes. Panel A summarizes the direction of effect, certainty of evidence, and number of contributing reviews. Panel B presents outcome-level classification according to effect direction and GRADE certainty. Bubble size and numbers adjacent to bubbles represent the number of representative reviews/meta-analyses contributing to each outcome. Interpretation should consider that evidence certainty reflects outcome-level GRADE assessment rather than methodological confidence of individual reviews. The outcome-level evidence map is shown in Figure 3.

**Figure 3:**
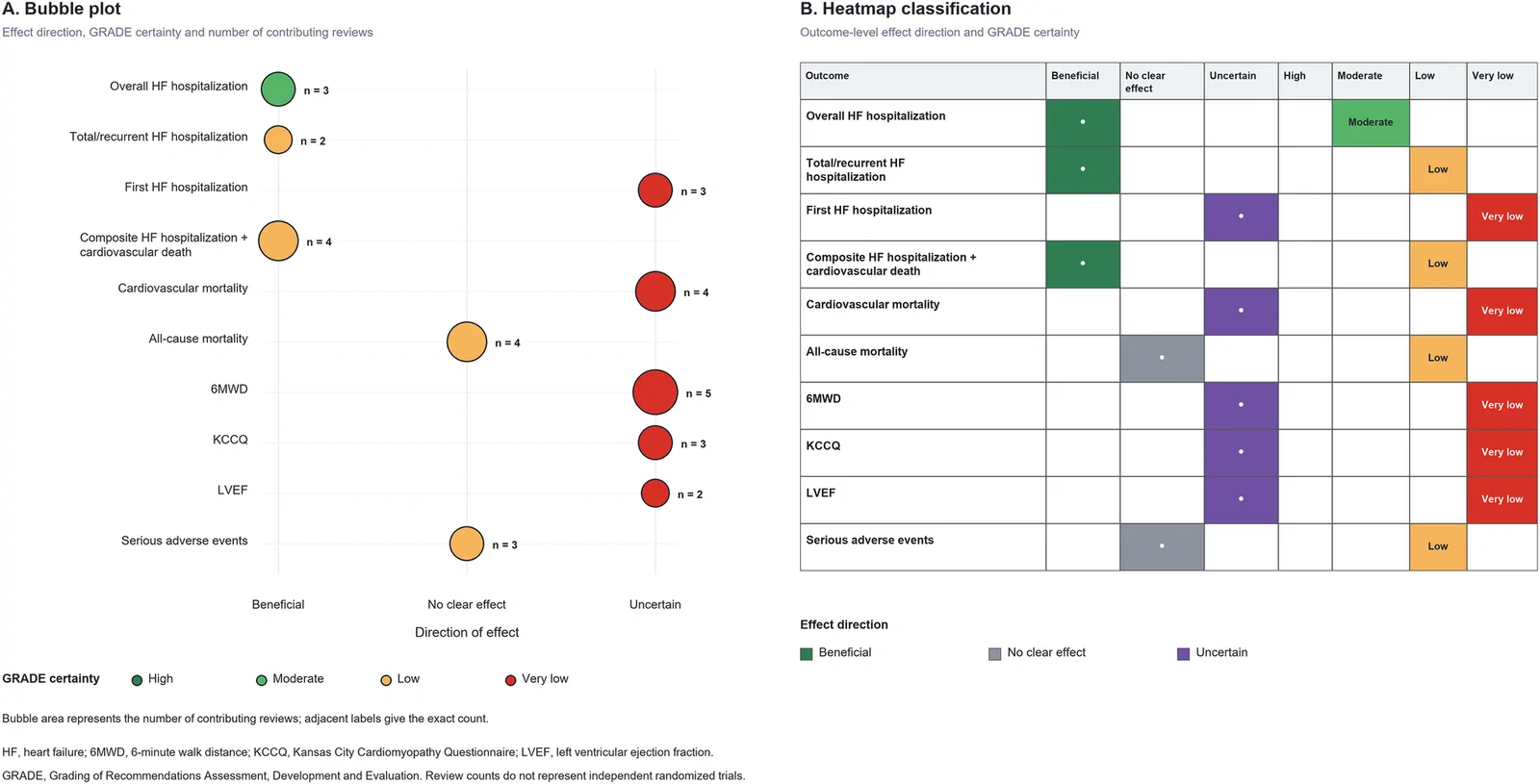
Evidence map of major outcomes of intravenous iron therapy in heart failure with iron deficiency. **(A)** Bubble plot showing effect direction, GRADE certainty, and the number of contributing reviews for each outcome. **(B)** Heatmap classification showing outcome-level effect direction and GRADE certainty. Bubble size represents the number of contributing reviews. Abbreviations: GRADE, Grading of Recommendations Assessment, Development and Evaluation; HF, heart failure; KCCQ, Kansas City Cardiomyopathy Questionnaire; LVEF, left ventricular ejection fraction; 6MWD, 6-minute walk distance.

Source review(s) indicates the representative systematic reviews/meta-analyses from which the corresponding effect estimates and evidence interpretations were derived. When multiple reviews reported similar outcomes, representative estimates were selected according to predefined criteria, including outcome definition alignment, clinical interpretability, contemporary evidence inclusion, and avoidance of duplicate representation of overlapping evidence. The outcome-specific evidence and corresponding GRADE certainty are summarized in Table 2.

**Table 2:** Summary of evidence by outcome.

Outcome	Direction of effect	Representative effect estimate (Outcome-specific metric)	Certainty of evidence (GRADE)	Interpretation	Source review(s) and corresponding estimate
Overall heart failure hospitalization	Reduced heart failure hospitalization	First HF admission RR 0.83 (95% CI 0.71–0.97); total HF admission rate ratio 0.81 (95% CI 0.74–0.89).	Moderate	Evidence suggests a potential reduction in heart failure hospitalization.	Graham et al.: first HF admission RR 0.83 (95% CI 0.71–0.97); Martens et al.: total HF admission rate ratio 0.81 (95% CI 0.74–0.89).
Total heart failure hospitalization	Reduced total/recurrent heart failure hospitalization	Total HF admissions rate ratio 0.739 (95% CI, 0.661–0.827), I2 = 75.6%; total HF hospitalizations pooled estimate 0.59 (95% CI, 0.40–0.88), with heterogeneity approaching 75–78%.	Low	Evidence suggests reduced total/recurrent heart failure hospitalization, but confidence is limited.	Martens et al.: total HF admissions rate ratio 0.739 (95% CI, 0.661–0.827), I2 = 75.6%; Padda et al.: total HF hospitalizations pooled estimate 0.59 (95% CI, 0.40–0.88).
First heart failure hospitalization	Uncertain effect on first heart failure hospitalization	First HF admission OR 0.855 (95% CI, 0.744–0.983), I2 = 60.3%; RR 0.83 (95% CI, 0.71–0.97); other first-HFH estimates crossed the null.	Very Low	The effect is very uncertain; interpret cautiously.	Ahmed et al.: first HF admission OR 0.855 (95% CI, 0.744–0.983), I2 = 60.3%; Graham et al.: first HF admission RR 0.83 (95% CI, 0.71–0.97); Tanashat et al.: first-HFH estimates with confidence intervals crossing the null.
Composite heart failure hospitalization + cardiovascular death	Reduced composite outcome of heart failure hospitalization and cardiovascular death	Composite estimates favored IV iron/FCM, with pooled estimates ranging from 0.79 to 0.89 across reviews.	Low	Evidence suggests a reduction in the composite outcome of heart failure hospitalization and cardiovascular death.	Taha et al., Mhanna et al., and Mohamed et al.: pooled composite estimates favored IV iron/FCM and ranged from 0.79 to 0.89.
Cardiovascular mortality	Uncertain effect on cardiovascular mortality	Risk ratio or odds ratio estimates were approximately 0.87–0.90, with several confidence intervals crossing the null.	Very Low	The effect is very uncertain; interpret cautiously.	Graham et al., Martens et al., Shalabi et al., and Ahmed et al.: cardiovascular mortality estimates generally ranged around RR/OR 0.87–0.90, with inconsistent statistical significance.
All-cause mortality	No clear effect on all-cause mortality	Risk ratio or odds ratio estimates were approximately 0.90–0.95 and generally close to the null.	Low	Evidence suggests no clear effect on all-cause mortality, but confidence is limited.	Qian et al., Vukadinovic et al., Wang et al., and Mhanna et al.: all-cause mortality estimates were generally close to the null, approximately RR/OR 0.90–0.95.
6MWD	Uncertain effect on 6MWD	Improvements in 6MWD were approximately 29–31 m in two reviews; one review reported a smaller mean difference of 14.03 m with confidence intervals crossing the null.	Very Low	The effect is very uncertain; interpret cautiously.	Taha et al. and Pintaningrum and Yunandari: 6MWD improvements of approximately 29–31 m; Padda et al.: smaller mean difference of 14.03 m with confidence intervals crossing the null.
KCCQ	Uncertain effect on Kansas City Cardiomyopathy Questionnaire scores	Variable effects were reported; some analyses suggested improvement, whereas others showed no statistically significant difference.	Very Low	The effect is very uncertain; interpret cautiously.	Taha et al. and Padda et al.: variable KCCQ effects; some analyses suggested improvement, whereas others showed no statistically significant difference.
LVEF	Uncertain effect on LVEF	Mean differences were approximately 2.7% and 4.54%, with imprecise confidence intervals and uncertain clinical significance.	Very Low	The effect is very uncertain; interpret cautiously.	Mhanna et al.: LVEF mean difference approximately 2.7%; Padda et al.: LVEF mean difference approximately 4.54%.
Serious adverse events	No clear effect on serious adverse events	Serious adverse events RR, 0.98 (95% CI, 0.93–1.03); safety estimate 0.73 (95% CI, 0.49–1.10).	Low	No clear increase in serious adverse events was identified, although certainty remains limited.	Rangwala et al.: serious adverse events RR, 0.98 (95% CI, 0.93–1.03); Padda et al.: safety estimate 0.73 (95% CI 0.49–1.10).

GRADE, grading of recommendations assessment, development and evaluation; HF, heart failure; KCCQ, Kansas City Cardiomyopathy Questionnaire; LVEF, left ventricular ejection fraction; 6MWD, 6 min walk distance; IV, intravenous; FCM, ferric carboxymaltose.

## Discussion

Synthesizing these data reveals a relatively localized primary finding: review-level evidence most consistently supports a potential reduction in heart failure hospitalizations with intravenous iron therapy, whereas its therapeutic efficacy regarding cardiovascular or all-cause mortality, functional capacity, health status, and LVEF remains far less conclusive. This disparity underscores that the clinical benefits of intravenous iron are not uniformly established across all clinical and surrogate endpoints. The review-level evidence requires cautious interpretation. All 18 representative reviews were rated as low or critically low confidence by AMSTAR2 (39), and many conclusions were derived from primary trials with substantial overlap. Only 23 unique randomized trials contributed to the core evidence base, while these trials appeared 163 times across reviews, resulting in a CCA of 35.81%. Therefore, the apparent abundance of published reviews likely overestimates the amount of independent evidence. The prioritization of 18 representative reviews from the 45 eligible reviews was performed to minimize redundant synthesis of highly overlapping evidence while preserving the broader eligible evidence base.

Crucially, the methodological quality of the synthesized reviews and the certainty of specific clinical outcomes represent distinct dimensions of evidence credibility. Although all prioritized reviews were characterized by low or critically low confidence under AMSTAR 2, the heart failure hospitalization outcome was consistently supported across reviews and backed by large-scale randomized trials. Consequently, the moderate GRADE certainty assigned to this endpoint reflects the consistency and precision of the outcome-level data itself, rather than implying an absence of methodological flaws in the secondary literature. The rating was capped at moderate because review-level deficiencies, extensive trial overlap, and discordant hospitalization definitions persist as key sources of residual uncertainty. The hospitalization signal is clinically plausible. Iron is involved in mitochondrial respiration, oxidative metabolism, skeletal-muscle energetics, and myocardial energy production, in addition to its role in hemoglobin synthesis (4, 7, 44). In patients with heart failure, iron deficiency may impair peripheral oxygen utilization and reduce exercise tolerance even in the absence of overt anemia. Restoration of iron availability through intravenous administration may improve functional reserve and reduce susceptibility to clinical deterioration, providing a potential explanation for the more consistent hospitalization findings.

Crucially, mitigating hospitalization does not guarantee a concomitant reduction in mortality. Survival in heart failure patients is governed by a multitude of competing risks (11, 13)—such as age, renal insufficiency, ischemic heart disease, arrhythmic burden, comorbidities, and baseline guideline-directed medical therapy—which collectively dilute the observable impact of iron repletion on hard survival endpoints. This complex interplay may help explain why the evidence for hospitalization reduction has been more consistent than that for a mortality benefit. Moreover, the lack of a consistent signal for LVEF improvement leaves effects on resting left ventricular systolic recovery uncertain; peripheral and metabolic mechanisms may contribute to the observed hospitalization findings. These findings should also be interpreted in the context of the evolving clinical trial evidence. Earlier studies, including FAIR-HF, CONFIRM-HF, and EFFECT-HF, primarily focused on symptoms, functional capacity, and quality of life (18–20), whereas subsequent trials increasingly evaluated clinical events. AFFIRM-AHF demonstrated fewer heart failure hospitalization events after acute heart failure stabilization without a clear cardiovascular mortality benefit (21). IRONMAN expanded the evidence base for ferric derisomaltose and recurrent-event outcomes (22). HEART-FID and FAIR-HF2 provided larger contemporary datasets but did not establish a clear mortality advantage (23, 24). Collectively, the trial trajectory supports hospitalization reduction as the most reproducible signal, while mortality and remodeling outcomes remain unresolved.

The choice of iron formulation is important for clinical interpretation. Most available evidence is derived from intravenous ferric carboxymaltose, including FAIR-HF, CONFIRM-HF, EFFECT-HF, AFFIRM-AHF, HEART-FID, and FAIR-HF2. Evidence for ferric derisomaltose is more limited and is mainly derived from IRONMAN and subsequent reviews incorporating this trial. Earlier iron sucrose studies were generally smaller and contributed less contemporary outcome evidence. Although some eligible reviews included mixed iron strategies or oral iron comparisons, these findings should not be interpreted as equivalent evidence for intravenous iron efficacy. Oral iron differs from intravenous formulations in bioavailability and iron delivery kinetics, and IRONOUT-HF did not demonstrate improvement in exercise capacity with oral iron (45). Therefore, the conclusions of this umbrella review primarily reflect intravenous iron therapy rather than iron supplementation as a broad therapeutic category.

Because most available evidence was derived from ferric carboxymaltose and direct comparative evidence between intravenous iron formulations remains limited, findings were interpreted primarily as class-level effects rather than evidence of superiority of any specific formulation.

The most consistent review-level signal concerns heart failure hospitalization. Evidence supporting reductions in mortality, cardiac remodeling, and durable functional improvement remains less certain. This distinction is clinically relevant because a therapy may reasonably reduce hospitalization risk without demonstrating a comparable effect on survival or structural cardiac outcomes.

Our findings are broadly aligned with contemporary international guideline recommendations, while also helping to define the boundaries of the underlying evidence. The 2022 AHA/ACC/HFSA guideline assigns intravenous iron replacement a Class IIa recommendation in patients with HFrEF and iron deficiency, with or without anemia, to improve functional status and quality of life. For symptomatic patients with HFrEF or HFmrEF and iron deficiency, the 2023 ESC focused update recommends intravenous iron for symptom and quality-of-life improvement (Class I, Level A), and supports consideration of ferric carboxymaltose or ferric derisomaltose for reducing heart failure hospitalization risk (Class IIa, Level A) (12, 13). In the present umbrella review, the most consistent review-level signal concerned heart failure hospitalization, whereas mortality benefit remained uncertain and functional or health-status outcomes were heterogeneous and often supported by low or very low certainty evidence. This pattern is consistent with guideline emphasis on selected clinical benefits, but it should not be interpreted as class-wide support across all heart failure phenotypes.

Importantly, the randomized evidence contributing to both current guideline recommendations and the present umbrella review was concentrated predominantly in patients with reduced ejection fraction, with some contemporary trials extending eligibility into the mildly reduced ejection fraction range. Evidence specifically derived from HFpEF remains limited. Because randomized evidence was derived predominantly from patients with reduced or mildly reduced LVEF, the findings should not be directly generalized to patients with preserved ejection fraction. Treatment decisions should therefore remain individualized and should consider heart failure phenotype, ferritin and transferrin saturation levels, renal function, recent hospitalization history, background guideline-directed medical therapy, comorbidities, clinical setting, and availability of specific iron formulations. Based on the current review-level evidence, the most consistent evidence of a potential clinical benefit of intravenous iron concerns overall heart failure hospitalization, whereas a mortality-modifying effect has not been established.

This umbrella review has several strengths. We searched major international and Chinese databases and integrated multiple methodological approaches, including AMSTAR2, GRADE, CCA analysis, and evidence mapping. This allowed assessment not only of treatment effects but also of methodological confidence, certainty of evidence, direction of effect, and redundancy across reviews. The prioritization of representative reviews was designed to reduce repeated synthesis of overlapping evidence while maintaining transparency regarding the broader eligible literature (55).

Although CCA demonstrated substantial overlap among the prioritized reviews, this estimate should not be generalized to all eligible reviews because it was calculated within the evidence set included in the quantitative synthesis. The prioritization strategy may have influenced the quantitative synthesis; however, the qualitative concordance assessment across all eligible reviews suggested that inclusion of the broader review landscape would unlikely alter the principal conclusions.

Another limitation is that heart failure phenotype was not uniformly defined or reported across the contributing reviews and underlying trials. LVEF eligibility thresholds varied, and several older studies predated contemporary HFrEF, HFmrEF, and HFpEF classifications. Therefore, phenotype-specific comparative conclusions could not be established, and extrapolation to HFpEF should be made cautiously. Rather than viewing intravenous iron as a universally established class-wide therapy for all heart failure settings, clinical application should be framed around the specific clinical phenotypes, iron formulations, and care settings validated in large-scale trials. Current evidence suggests a potential reduction in overall heart failure hospitalization in selected patients with heart failure, predominantly those with reduced LVEF and with some trials extending eligibility into the mildly reduced EF range; interpretation is constrained by high trial overlap, review-level methodological deficiencies, and varying hospitalization definitions.

Future evidence synthesis should move beyond repeated pooling of the same trial base. More informative approaches would include formulation-specific analyses separating ferric carboxymaltose from ferric derisomaltose, setting-specific analyses distinguishing acute decompensated from stable chronic heart failure, and consistent reporting of first vs. recurrent hospitalization outcomes. Future trials and reviews should also incorporate post-treatment iron status, renal-function strata, sex-specific effects, heart failure phenotype, and background guideline-directed medical therapy to improve clinical interpretation and guideline implementation.

## Conclusion

To conclude, cumulative review-level evidence, derived predominantly from patients with reduced LVEF and with some trials extending into the mildly reduced EF range, probably supports a reduction in heart failure hospitalization with intravenous iron in patients with iron deficiency. This pattern is broadly consistent with contemporary AHA/ACC/HFSA and ESC guideline recommendations. However, the available evidence should not be generalized across the entire heart failure spectrum, particularly to HFpEF, for which dedicated randomized evidence remains limited. Class-wide generalizations across intravenous iron formulations and clinical contexts are further constrained by extensive trial overlap, methodological limitations of the contributing reviews, and inconsistent outcome definitions. The effects on survival, long-term functional status, and cardiac remodeling remain uncertain.

## Data Availability

The original contributions presented in the study are included in the article/Supplementary Material, further inquiries can be directed to the corresponding author.

## References

[1] Klip IJT Comin-Colet J Voors AA Ponikowski P Enjuanes C Banasiak W. Iron deficiency in chronic heart failure: an international pooled analysis. Am Heart J. (2013) 165(4):575–582.e3. 10.1016/j.ahj.2013.01.017 23537975

[2] Jankowska EA Rozentryt P Witkowska A Nowak J Hartmann O Ponikowska B. Iron deficiency: an ominous sign in patients with systolic chronic heart failure. Eur Heart J. (2010) 31(15):1872–80. 10.1093/eurheartj/ehq158 20570952

[3] Anand IS Gupta P. Anemia and iron deficiency in heart failure. Circulation. (2018) 138(1):80–98. 10.1161/circulationaha.118.030099 29967232

[4] Okonko DO Mandal AKJ Missouris CG Poole-Wilson PA. Disordered iron homeostasis in chronic heart failure. J Am Coll Cardiol. (2011) 58(12):1241–51. 10.1016/j.jacc.2011.04.040 21903058

[5] Von Haehling S Jankowska EA Van Veldhuisen DJ Ponikowski P Anker SD. Iron deficiency and cardiovascular disease. Nat Rev Cardiol. (2015) 12(11):659–69. 10.1038/nrcardio.2015.109 26194551

[6] Jankowska EA Von Haehling S Anker SD Macdougall IC Ponikowski P. Iron deficiency and heart failure: diagnostic dilemmas and therapeutic perspectives. Eur Heart J. (2013) 34(11):816–29. 10.1093/eurheartj/ehs224 23100285 PMC3596759

[7] Van Der Meer P Van Der Wal HH Melenovsky V. Mitochondrial function, skeletal muscle metabolism, and iron deficiency in heart failure. Circulation. (2019) 139(21):2399–402. 10.1161/circulationaha.119.040134 31107619

[8] Comin-Colet J Lainscak M Dickstein K Filippatos GS Johnson P Lüscher TF. The effect of intravenous ferric carboxymaltose on health-related quality of life in patients with chronic heart failure and iron deficiency: a subanalysis of the FAIR-HF study. Eur Heart J. (2013) 34(1):30–8. 10.1093/eurheartj/ehr504 22297124 PMC3533918

[9] De Biase N Del Punta L L'Hoyes W Pellicori P Cleland JGF Masini G. Associations of iron deficiency with cardiac function, congestion, exercise capacity and prognosis in heart failure. Eur J Heart Fail. (2025) 27(5):889–900. 10.1002/ejhf.3534 39607281

[10] Martens P Tang WHW. Defining iron deficiency in heart failure: importance of transferrin saturation. Circ Heart Fail. (2024) 17(4):e011440. 10.1161/circheartfailure.123.011440 38567517

[11] McDonagh TA Metra M Adamo M Gardner RS Baumbach A Böhm M. 2021 ESC guidelines for the diagnosis and treatment of acute and chronic heart failure. Eur Heart J. (2021) 42(36):3599–726. 10.1093/eurheartj/ehab368 34447992

[12] McDonagh TA Metra M Adamo M Gardner RS Baumbach A Böhm M. 2023 Focused update of the 2021 ESC guidelines for the diagnosis and treatment of acute and chronic heart failure. Eur Heart J. (2023) 44(37):3627–39. 10.1093/eurheartj/ehad195 37622666

[13] Heidenreich PA Bozkurt B Aguilar D Allen LA Byun JJ Colvin MM. 2022 AHA/ACC/HFSA guideline for the management of heart failure: a report of the American College of Cardiology/American Heart Association joint committee on clinical practice guidelines. Circulation. (2022) 145(18):e895–1032. 10.1161/cir.0000000000001063 35363499

[14] Lam CSP Doehner W Comin-Colet J. Iron deficiency in chronic heart failure: case-based practical guidance. ESC Heart Fail. (2018) 5(5):764–71. 10.1002/ehf2.12333 30073785 PMC6165963

[15] Bolger AP Bartlett FR Penston HS O’Leary J Pollock N Kaprielian R. Intravenous iron alone for the treatment of anemia in patients with chronic heart failure. J Am Coll Cardiol. (2006) 48(6):1225–7. 10.1016/j.jacc.2006.07.015 16979010

[16] Toblli JE Lombraña A Duarte P Di Gennaro F. Intravenous iron reduces NT-pro-brain natriuretic peptide in anemic patients with chronic heart failure and renal insufficiency. J Am Coll Cardiol. (2007) 50(17):1657–65. 10.1016/j.jacc.2007.07.029 17950147

[17] Okonko DO Grzeslo A Witkowski T Mandal AKJ Slater RM Roughton M. Effect of intravenous iron sucrose on exercise tolerance in anemic and nonanemic patients with symptomatic chronic heart failure and iron deficiency. J Am Coll Cardiol. (2008) 51(2):103–12. 10.1016/j.jacc.2007.09.036 18191732

[18] Anker SD Comin Colet J Filippatos G Willenheimer R Dickstein K Drexler H. Ferric carboxymaltose in patients with heart failure and iron deficiency. N Engl J Med. (2009) 361(25):2436–48. 10.1056/nejmoa0908355 19920054

[19] Ponikowski P Van Veldhuisen DJ Comin-Colet J Ertl G Komajda M Mareev V. Beneficial effects of long-term intravenous iron therapy with ferric carboxymaltose in patients with symptomatic heart failure and iron deficiency. Eur Heart J. (2015) 36(11):657–68. 10.1093/eurheartj/ehu385 25176939 PMC4359359

[20] van Veldhuisen DJ Ponikowski P Van Der Meer P Metra M Böhm M Doletsky A. Effect of ferric carboxymaltose on exercise capacity in patients with chronic heart failure and iron deficiency. Circulation. (2017) 136(15):1374–83. 10.1161/circulationaha.117.027497 28701470 PMC5642327

[21] Ponikowski P Kirwan B-A Anker SD McDonagh T Dorobantu M Drozdz J. Ferric carboxymaltose for iron deficiency at discharge after acute heart failure: a multicentre, double-blind, randomised, controlled trial. Lancet. (2020) 396(10266):1895–904. 10.1016/s0140-6736(20)32339-4 33197395

[22] Kalra PR Cleland JGF Petrie MC Thomson EA Kalra PA Squire IB. Intravenous ferric derisomaltose in patients with heart failure and iron deficiency in the UK (IRONMAN): an investigator-initiated, prospective, randomised, open-label, blinded-endpoint trial. Lancet. (2022) 400(10369):2199–209. 10.1016/s0140-6736(22)02083-9 36347265

[23] Mentz RJ Garg J Rockhold FW Butler J De Pasquale CG Ezekowitz JA. Ferric carboxymaltose in heart failure with iron deficiency. N Engl J Med. (2023) 389(11):975–86. 10.1056/nejmoa2304968 37632463

[24] Anker SD Friede T Butler J Talha KM Placzek M Diek M. Intravenous ferric carboxymaltose in heart failure with iron deficiency. JAMA. (2025) 333(22):1965. 10.1001/jama.2025.3833 40159390 PMC11955906

[25] Qian C Wei B Ding J Wu H Wang Y. The efficacy and safety of iron supplementation in patients with heart failure and iron deficiency: a systematic review and meta-analysis. Can J Cardiol. (2016) 32(2):151–9. 10.1016/j.cjca.2015.06.009 26454467

[26] Vukadinović D Abdin A Emrich I Schulze PC Von Haehling S Böhm M. Efficacy and safety of intravenous iron repletion in patients with heart failure: a systematic review and meta-analysis. Clin Res Cardiol. (2023) 112(7):954–66. 10.1007/s00392-023-02207-2 PMC10116902 37074386

[27] Graham FJ Pellicori P Kalra PR Ford I Bruzzese D Cleland JGF. Intravenous iron in patients with heart failure and iron deficiency: an updated meta-analysis. Eur J Heart Fail. (2023) 25(4):528–37. 10.1002/ejhf.2810 36823953 PMC10946839

[28] Martens P Augusto SN Mullens W Tang WHW. Meta-analysis and metaregression of the treatment effect of intravenous iron in iron-deficient heart failure. JACC Heart Fail. (2024) 12(3):525–36. 10.1016/j.jchf.2023.11.006 38069996

[29] Ahmed M Zulfiqar E Hashmi TM Zia R Ashraf H Naveed MA. Intravenous ferric carboxymaltose in patients with heart failure and iron deficiency: a systematic review and meta-analysis of randomized controlled trials with trial sequential analysis. ESC Heart Fail. (2026) 13(1):xvaf018. 10.1093/eschf/xvaf018 41711738 PMC13108314

[30] Sciatti E Nesti U Di Lenarda A. Indirect comparison between ferric carboxymaltose and oral iron replacement in heart failure with reduced ejection fraction: a network meta-analysis. Monaldi Arch Chest Dis. (2021) 91:1703. 10.4081/monaldi.2021.1703 33794590

[31] Khan MS Usman MS Von Haehling S Doehner W Stewart Coats AJ. Ferric carboxymaltose for the treatment of iron-deficient heart failure patients: a systematic review and meta-analysis. ESC Heart Fail. (2020) 7(6):3392–400. 10.1002/ehf2.13146 33586856 PMC7754952

[32] Taha AM Elsaeidy AS Nada SA Sharma S Ghonaim MM Ahsan A. Efficacy of intravenous ferric carboxymaltose in heart failure patients with iron deficiency anemia: a meta-analysis of 6271 patients. Clin Drug Investig. (2024) 44(12):879–96. 10.1007/s40261-024-01401-y 39527395 PMC11638377

[33] Rangwala BS Zuhair V Mustafa MS Mussarat A Khan AW Danish F. Ferric carboxymaltose for iron deficiency in patients with heart failure: a systematic review and meta-analysis. Future Sci OA. (2024) 10(1):2367956. 10.1080/20565623.2024.2367956 38982752 PMC11238921

[34] Padda I Sebastian SA Fabian D Sethi Y Johal G. The efficacy and safety of ferric carboxymaltose in heart failure with reduced ejection fraction and iron deficiency: an updated systematic review and meta-analysis of randomized controlled trials. Diseases. (2024) 12(12):339. 10.3390/diseases12120339 39727669 PMC11727542

[35] Page MJ McKenzie JE Bossuyt PM Boutron I Hoffmann TC Mulrow CD. The PRISMA 2020 statement: an updated guideline for reporting systematic reviews. Br Med J. (2021) 372:n71. 10.1136/bmj.n71 PMC8005924 33782057

[36] Moliner P Comin-Colet J. Iron deficiency and supplementation therapy in heart failure. Nat Rev Cardiol. (2022) 19(9):571–2. 10.1038/s41569-022-00747-0 35788563

[37] Pieper D Antoine SL Mathes T Neugebauer EAM Eikermann M. Systematic review finds overlapping reviews were not mentioned in every other overview. J Clin Epidemiol. (2014) 67(4):368–75. 10.1016/j.jclinepi.2013.11.007 24581293

[38] Hennessy EA Johnson BT. Examining overlap of included studies in meta-reviews: guidance for using the corrected covered area index. Res Synth Methods. (2020) 11(1):134–45. 10.1002/jrsm.1390 31823513 PMC8555740

[39] Shea BJ Reeves BC Wells G Thuku M Hamel C Moran J. AMSTAR 2: a critical appraisal tool for systematic reviews that include randomised or non-randomised studies of healthcare interventions, or both. Br Med J. (2017) 358:j4008. 10.1136/bmj.j4008 PMC5833365 28935701

[40] Guyatt G Oxman AD Akl EA Kunz R Vist G Brozek J. GRADE guidelines: 1. Introduction—gRADE evidence profiles and summary of findings tables. J Clin Epidemiol. (2011) 64(4):383–94. 10.1016/j.jclinepi.2010.04.026 21195583

[41] Tanashat M Abouzid M Khlidj Y Abuelazm M Ramadan A Altobaishat O. Efficacy and safety of intravenous iron therapy in heart failure patients with iron deficiency: a systematic review and meta-analysis of randomized controlled trials. J Med Sci. (2024) 93(4):e1183. 10.20883/medical.e1183

[42] Mhanna M Sauer MC Al-Abdouh A Jabri A Beran A Barbarawi M. Intravenous iron therapy for patients with iron deficiency and heart failure: a systematic review and meta-analysis of randomized controlled trials. Bayl Univ Med Cent Proc. (2024) 37(3):466–76. 10.1080/08998280.2024.2326387 PMC11018061 38628339

[43] Mohamed AE Gadelmawla AF Kholeif Z Balbaa E Awashra A Elnady M. Ferric carboxymaltose among patients with heart failure and iron deficiency: an updated meta-analysis of randomized controlled trials. Cardiovasc Toxicol. (2026) 26(1):15. 10.1007/s12012-026-10090-2 41524998

[44] Melenovsky V Hlavata K Sedivy P Dezortova M Borlaug BA Petrak J. Skeletal muscle abnormalities and iron deficiency in chronic heart failure. Circ Heart Fail. (2018) 11(9):e004800. 10.1161/circheartfailure.117.004800 30354361

[45] Lewis GD Malhotra R Hernandez AF McNulty SE Smith A Felker GM. Effect of oral iron repletion on exercise capacity in patients with heart failure with reduced ejection fraction and iron deficiency. JAMA. (2017) 317(19):1958. 10.1001/jama.2017.5427 28510680 PMC5703044

[46] Ahmed M Shafiq A Javaid H Singh P Shahbaz H Maniya MT. Intravenous iron therapy for heart failure and iron deficiency: an updated meta-analysis of randomized clinical trials. ESC Heart Fail. (2025) 12(1):43–53. 10.1002/ehf2.14905 38965691 PMC11769671

[47] Anker SD Kirwan B-A Van Veldhuisen DJ Filippatos G Comin-Colet J Ruschitzka F. Effects of ferric carboxymaltose on hospitalisations and mortality rates in iron-deficient heart failure patients: an individual patient data meta-analysis. Eur J Heart Fail. (2018) 20(1):125–33. 10.1002/ejhf.823 28436136

[48] Aromataris E Fernandez R Godfrey CM Holly C Khalil H Tungpunkom P. Summarizing systematic reviews. Int J Evid Based Healthc. (2015) 13(3):132–40. 10.1097/xeb.0000000000000055 26360830

[49] Marques P Vasques-Nóvoa F Matias P Vieira JT Mavrakanas TA Sharma A. Influence of iron deficiency definition on the efficacy of intravenous iron in heart failure: a meta-analysis of randomized trials. Clin Res Cardiol. (2024). [Online ahead of print]. 10.1007/s00392-024-02557-5 39432015

[50] Martens P Dupont M Dauw J Nijst P Herbots L Dendale P. The effect of intravenous ferric carboxymaltose on cardiac reverse remodelling following cardiac resynchronization therapy—the IRON-CRT trial. Eur Heart J. (2021) 42(48):4905–14. 10.1093/eurheartj/ehab411 34185066 PMC8691806

[51] Mototani R Watanabe A Kuno T Briasoulis A. Effect of intravenous iron-carbohydrate complexes in patients with heart failure with reduced ejection fraction and iron deficiency: a meta-analysis of randomized controlled trials. Hellenic J Cardiol. (2023) 72:67–9. 10.1016/j.hjc.2023.03.005 36958646

[52] Núñez J Miñana G Cardells I Palau P Llàcer P Fácila L. Noninvasive imaging estimation of myocardial iron repletion following administration of intravenous iron: the myocardial-IRON trial. J Am Heart Assoc. (2020) 9(4):e014254. 10.1161/jaha.119.014254 PMC7070181 32067585

[53] Pintaningrum Y Yunandari NP. Iron therapy effect on chronic heart failure and iron-deficiency anemia: a systematic review and meta-analysis. Egypt Heart J. (2024) 76(1):116. 10.1186/s43044-024-00545-8 39214928 PMC11364828

[54] Shalabi L Ibrahim A Ramadan S Zreigh S Shalabi S Rifai M. Intravenous ferric carboxymaltose for iron deficiency in heart failure patients: a meta-analysis with trial sequential analysis. Eur J Pharmacol. (2025) 1008:178314. 10.1016/j.ejphar.2025.178314 41177386

[55] Siontis KC Ioannidis JPA. Replication, duplication, and waste in a quarter million systematic reviews and meta-analyses. Circ Cardiovasc Qual Outcomes. (2018) 11(12):e005212. 10.1161/circoutcomes.118.005212 30562075

[56] Wang H Li Y Zhou J Wang J Shao J Yue S. Impact of intravenous iron in patients with heart failure and iron deficiency: an updated meta-analysis of randomized controlled trials. BMC Cardiovasc Disord. (2024) 24(1):695. 10.1186/s12872-024-04368-y 39627692 PMC11616113

